# COVID 19 infection and mucormycosis—a dangerously increasing combination

**DOI:** 10.1186/s43163-021-00121-w

**Published:** 2021-05-27

**Authors:** Satvinder Singh Bakshi, Vinoth Kumar Kalidoss

**Affiliations:** 1 Department of ENT and Head & Neck Surgery, AIIMS Mangalagiri, Guntur, Andhra Pradesh 522503 India; 2 Department of Community and Family Medicine, AIIMS Mangalagiri, Guntur, Andhra Pradesh 522503 India

**Keywords:** COVID 19, Mucormycosis, Glucocorticoids

To the editor,

## Background

With the current pandemic of coronavirus disease 2019 (COVID-19) raging, there has been a frantic search for treatment options. Out of the several treatment options that have been tried with varying degrees of success, systemic glucocorticoids have been shown to improve survival in COVID-19. Unfortunately, the widespread use of glucocorticoids has also led to an increase in the side effects of these drugs especially an increase in secondary bacterial or fungal infections. COVID-19-associated pulmonary aspergillosis (CAPA) complicating the course of COVID-19 has widely been reported; there are only a few reports on mucormycosis [1, 2], probably due to the lack of clinical suspicion and difficulty in isolation of causative fungi.

## Main text

Mucormycosis is an acute or subacute rapidly progressing infections caused by the angioinvasive fungi in the order of Mucorales [3]. It most commonly affects patients with poorly controlled diabetes mellitus and immunocompromised patients, leading to significant morbidity and mortality. The most common region affected is the nose, paranasal sinus, and brain leading to rhino-orbital and rhino-cerebral mucormycosis; this is also seen in the present pandemic and most of the case reports are of rhino-orbito-cerebral mucormycosis [4]; however, there are reports of pulmonary [1] and gastrointestinal mucormycosis too [5]. The cause for increased mucor infection in patients is complex and includes an interplay of factors, like pre-existing diseases, such as diabetes mellitus; use of immunosuppressive therapy like glucocorticoids and tocilizumab; pre-existing lung conditions; and systemic immune alterations by the virus itself like reduced number of T lymphocytes, CD4+T, and CD8+T cells [4].

Many challenges have also emerged in the management of mucormycosis during this pandemic. Early detection, control of hyperglycemia, liposomal amphotericin B, and surgical debridement are the cornerstones in the successful management of mucormycosis. However, in the current scenario, it is difficult to control the hyperglycemia due to the extensive use of steroids, most operation theaters remain shut due to diversion of manpower and other resources to other departments; therefore, delaying surgical debridement and the presence of coexisting multi-organ dysfunction in these patients makes it difficult to shift them for imaging studies.

## Conclusion

In this scenario, it is imperative that clinicians should be sensitized to the increased risk of development of this fatal infection, especially while treating diabetic coronavirus-affected patients with systemic steroids. In addition, judicious use of glucocorticoids must be advocated in all patients.

## Data Availability

Not applicable

## Abbreviations

COVID-19: Corona virus 2019
CAPA: COVID-19-associated pulmonary aspergillosis
